# Fatigue in adults with post-infectious fatigue syndrome: a qualitative content analysis

**DOI:** 10.1186/s12912-015-0115-5

**Published:** 2015-11-28

**Authors:** Eva Stormorken, Leonard A. Jason, Marit Kirkevold

**Affiliations:** 1 Department of Nursing Science, Institute of Health and Society, University of Oslo, P.O.B. 1130, Blindern, 0318 Oslo Norway; 2 Center for Community Research, DePaul University, 990 W. Fullerton Ave, Suite 3100, Chicago, Illinois 60614 USA

**Keywords:** Adult patients, Fatigue, In-depth interview, Myalgic encephalomyelitis chronic fatigue syndrome, Nursing qualitative research, Qualitative research, Patient experiences, Post-infectious fatigue syndrome

## Abstract

**Background:**

Fatigue is a major problem among individuals with post-infectious fatigue syndrome (PIFS), also known as chronic fatigue syndrome or myalgic encephalomyelitis. It is a complex phenomenon that varies across illnesses. From a nursing perspective, knowledge and understanding of fatigue in this illness is limited. Nurses lack confidence in caring for these patients and devalue their professional role. The aim of this study was to explore in-depth the experiences of fatigue among individuals with PIFS. A detailed description of the phenomenon of fatigue is presented. Increased knowledge would likely contribute to more confident nurses and improved nursing care.

**Methods:**

A qualitative study with open interviews was employed. In-depth interviews with patients were fully transcribed and underwent a qualitative content analysis. A maximum variation sample of 26 affected adults between 26–59 years old was recruited from a population diagnosed at a fatigue outpatient clinic.

**Results:**

The fatigue was a post-exertional, multidimensional, fluctuating phenomenon with varying degrees of severity and several distinct characteristics and was accompanied by concomitant symptoms. Fatigue was perceived to be an all-pervasive complex experience that substantially reduced the ability to function personally or professionally. A range of trigger mechanisms evoked or worsened the fatigue, but the affected were not always aware of what triggered it. There was an excessive increase in fatigue in response to even minor activities. An increase in fatigue resulted in the exacerbation of other concomitant symptoms. The term fatigue does not capture the participants’ experiences, which are accompanied by a considerable symptom burden that contributes to the illness experience and the severe disability.

**Conclusions:**

Although some aspects of the fatigue experience have been reported previously, more were added in our study, such as the dimension of awakening fatigue and the characteristic beyond time, when time passes unnoticed. We also identified trigger mechanisms such as emotional, neurological, social, financial, and pressure on oneself or from others. This in-depth exploration of fatigue in PIFS provides an overview of the dimensions, characteristics, and trigger mechanisms of fatigue, thus making better clinical observations, early recognition, improved communication with patients and more appropriate nursing interventions possible.

## Background

In 2004, 48,000 people were exposed to contaminated municipal drinking water in a part of Bergen, Norway. From the Norwegian Prescription Database, it was assumed that > 2500 persons had been affected by a gastrointestinal infection (GI) [1]. The parasite *Giardia lamblia* was found to be responsible, having entered the water supply through a leak in a sewer pipe [2]. The infection caused diarrhoea, weight loss, and other gastrointestinal complaints [1]. Affected people were treated with antibiotics, but a minority remained ill and severely fatigued. They were referred to the Outpatient Clinic at the Department of Neurology, Haukeland University Hospital from August 2005 to September 2007 with suspicion of post-infectious fatigue syndrome (PIFS) [3].

Fatigue is one of the most debilitating symptoms in PIFS and is required, among other symptoms, to meet the diagnostic criteria for myalgic encephalomyelitis (ME) or chronic fatigue syndrome (CFS), two other commonly used labels for this condition. The hallmarks of PIFS (ME/CFS) are fatigue, post-exertional malaise (PEM), pain, neurological complaints, autonomic and immune disturbances, and sleep difficulties that must have been present or recurrent for more than 6 months [4]. The patients’ functional level must be reduced by more than 50 % compared with pre-illness levels. The aetiology is elusive, and no cure currently exists. Few sufferers completely recover to pre-morbid health [5]. Afflicted persons are offered symptom alleviation, rehabilitation or educational services to learn to cope with the illness and improve function [6].

Fatigue is a mandatory symptom: “…*the patient must have a significant degree of new onset, unexplained, persistent, or recurrent physical and mental fatigue*” [4, 7]. It is difficult to capture because of its subjective and heterogeneous nature [8, 9] and is defined as “*a subjective unpleasant symptom which incorporates total body feelings ranging from tiredness to exhaustion creating an unrelenting overall condition which interferes with individuals’ ability to function to their normal capacity*” [10].

Only a few qualitative studies of fatigue have focused on its dimensions, specific characteristics, concomitant symptoms and trigger mechanisms. Fatigue has been grouped into physical and mental fatigue [11] and seen as a multidimensional phenomenon. Bennet et al. [12] identified 4 dimensions: fatigue, somatic symptoms, neurocognitive impairment, and mood disturbance, whilst Jason et al. [13] uncovered 5 dimensions: post-exertional, wired, brain fog, energy, and flu-like symptoms. PEM (symptom flare-up) is induced by physical activity, whereas wired fatigue is characterised by an excited physical and mental state, feeling tiredness and overstimulation simultaneously. PIFS fatigue also comprises lack of energy and stamina, prolonged restitution, disrupted sleep, and functional disability [13, 14]. Fatigue was not present at all times [15]. Two types of trigger mechanisms provoked symptom exacerbation: physical and mental exertions. Many participants had been unaware of what triggered increased fatigue and symptom exacerbations. The unpredictability of good and bad days resulted in the inability to control daily life [11]. The illness had inflicted a disruption between the body and the self [16]. The body had become an opposition to the self and was no longer trustworthy or reliable [17]. The desire to return to the pre-illness lifestyle conflicted with the need to take care of the ill body, having awareness of the body, and respecting its limits and needs [16]. Fatigue was perceived to be a whole-body experience in which body and self interacted [18].

Despite the above studies, PIFS-related fatigue is not completely understood or recognised [19]. Chew-Graham et al. [20] found that Practice Nurses had limited understanding of this illness and devalued their own skills. Furthermore, studies have found that approximately 50–60 % of fatigued patients with suspected PIFS (ME/CFS) instead had treatable conditions such as primary sleep disorders, psychiatric illnesses, chronic pain, or cardiovascular diseases [21, 22]. Only a few studies have explored the nature of fatigue in this illness in-depth and most of these have included participants without a verified infectious cause [23]. Qualitative studies with a broader and more holistic perspective on the nature of fatigue are lacking. Therefore, it would be valuable to explore fatigue in a well-defined group of patients with a known and diagnosed cause of PIFS. Limited knowledge among Registered Nurses (RNs) may result in a reduced ability to recognise the complexity or understand the nuances of fatigue in PIFS. This may cause inadequate nursing care or harm [24]. Consequently, the aim of this study was to explore the nature of post-infectious fatigue in a known population of PIFS following an outbreak of gastrointestinal infection caused by *G. lamblia*. More specifically, we sought to describe the dimensions, specific characteristics, concomitant symptoms and trigger mechanisms of fatigue in individuals with PIFS.

## Methods

### Methodology

This qualitative interview study had an exploratory design [25] and was approved by the Regional Committee for Medical and Health Research Ethics (REC, West) (No. 227.07) and Privacy Ombudsman for Research (No. 17416).

### Recruitment and sample

The post-giardiasis fatigued patients referred to the clinic were evaluated by a neurologist and diagnosed with PIFS according to international criteria for CFS [7]. In this PIFS cohort [3], the mean Fatigue Severity Score [26] was 6.64 (SD: 0.45). The subscores on the SF-36 [27] were significantly reduced, with the lowest scores for physical capacity, vitality and social functioning. The sample in this qualitative interview study was recruited from the cohort of 58 adults [3].

Because this patient group was considered heterogeneous [9], a purposive, maximum variation sample was recommended [28]. Requests for participation were distributed by mail from the clinic. Forty-four (76 %) patients responded and consented to participate. The selection procedure was explained in the request letter, and responders were told that not all would be contacted for an interview. A sample of 19 females and 7 males, 45 % of the PIFS cohort, was selected and contacted for an in-depth interview [25]. None of the participants dropped out. The gender distribution was consistent with population-based studies [29]. The demographic characteristics pertaining to gender, age and education level were in line with earlier findings at this clinic [30] and the *Giardia* outbreak [1]. The affected individuals had either visited or stayed in the part of the city supplied with the contaminated water, which was mainly a business area, with rental accommodations for students. Few old or young people were infected, and the only risk factor identified was drinking more than five glasses of water per day [1]. The sample characteristics and disability levels at different points in time are presented in Tables 1 and 2.

**Table 1:** Sample characteristics

Demographic variables		
Ethnicity		*N*
Caucasian		26 (100 %)
Age		Mean (range)
Total sample (*n* = 26)		40.9 (26–59)
Female (*n* = 19)		41.4 (26–59)
Male (*n* = 7)		39.6 (28–58)
Marital status		*N*
Single		12 (63.2 %)
Married		9 (10.5 %)
Cohabiting		2 (10.5 %)
Divorced		3 (15.8 %)
Household income^a^		*N*
Very low		3 (11.5 %)
Low		8 (30.8 %)
Average		9 (34.6 %)
High		5 (19.3 %)
Very high		1 (3.8 %)
Education years		*N*
Total sample (*n* = 26)		9–17+
Lower secondary education	≤9 years	2 (7.7 %)
Upper secondary education	10–13 years	4 (15.4 %)
College/university Bachelor’s	14–16 years	6 (23.1 %)
College/university postgraduate	≥17 years	14 (53.8 %)
Occupations^b^ and study status		*N*
Professional		11 (41 %)
Students		6 (23 %)
Skilled trades		3 (12 %)
Management		2 (8 %)
Associate professional and technical		1 (4 %)
Sales and customer service		1 (4 %)
Elementary occupations		1 (4 %)
*Missing*		1 (4 %)

^a^The five categories of household income were not assigned a numerical value and patients were asked to choose one subjectively

^b^[48]

**Table 2:** Disability levels at different points in time (*n* = 26)

Bell’s CFS disability scale^a^ score		Median (range)
Pre-*Giardia* infection	Spring 2004	100 (90–100)
At time of PIFS diagnosis	Point in time varied, unspecified	30 (10–70)
At time of being most disabled	Point in time varied, unspecified (missing: F = 1, M = 1)	20 (10–40)
When being ill for 3 years	Autumn 2007 (missing: F = 3)	40 (20–60)
At time of interview	Spring 2008	40 (10–100)^b^

^a^A 10-point disability grading scale ranging from 0–100. 0 **=** Severe symptoms on a continuous basis; bedridden constantly; unable to care for self. 100 = No symptoms with exercise; normal overall activity; able to work or do house/home work full-time with no difficulty [49, 50]. ^b^One participant’s score was 100 but had not resumed work to pre-illness level, was dependent on 50 % welfare benefits, and reported 51 symptoms and a reduced functional level

**Table 3:** Examples of meaning units, condensed meaning units and codes

Meaning unit	Condensed meaning unit	Code
Then came these symptoms that I could not stand; the noise—I could not tolerate light, could not bear the sensations at all.	Feeling of intolerances to noise, light and other sensations	Weird body
I’ve blacked it out. The period I was worst, I cannot say anything about… Yes, I felt that it was a sort of coma. Yes, time passed. It was just like the concept of time… disappeared completely. I had no concept of time.	The mind was in a sort of coma and the concept of time completely disappeared	Beyond time
The energy failure is so comprehensive. You are even tired of talking.	Talking exacerbates fatigue	Physical trigger

The participants had been engaged in either work or studies on a full-time basis pre-illness but were more or less unable to continue their pre-illness engagements after falling ill. Four years later, 61.5 % (13 females and 3 males) of the subjects were fully dependent on welfare benefits, and 38.5 % (6 females and 4 males) were 30–50 % part-time employed and partly dependent on welfare benefits. None had resumed full-time work or study.

The degree of severity and illness course varied (Table 2). Most but not all participants had improved somewhat after 4 years. The lowest disability level score was 10, indicating severe symptoms at rest, bedridden most of the time, no travel outside the house, and marked cognitive symptoms preventing concentration.

### Data collection

Face-to-face in-depth interviews were conducted 4 years after the subjects fell ill. Audio-recorded interviews took place in a dimly lit, quiet, and comfortably arranged wardroom at the hospital, the same day the participants had a scheduled appointment at the clinic. A topic list and an open-ended interview approach were used to ensure consistency. The topic list contained areas identified in previous research studies, medical textbooks on the subject and topics emerging from professional clinical experiences with people with the condition. The opening question was: ‘Please tell me about your experiences with the post-infectious fatigue’. Follow-up questions and prompts were used to elicit nuances and facets of the fatigue experience. Examples of follow-up questions: ‘How would you describe your fatigue to other people?’; ‘Tell me about your experiences’; or ‘Please tell me how you experience good and bad days.’ Prompts to obtain richer descriptions were used, such as ‘Can you provide more examples?’ or ‘What happened next?’

### Ethical consideration

The participants received written information about the study purpose and consented in writing to participate. Before the interview, they were asked again and verbally confirmed their consent. They were then informed of their right to decline from answering questions or withdraw at any time. The interviewer was aware of the distress the in-depth interview might provoke in this vulnerable group. Renewal of informed consent was obtained in instances of strong emotional feelings, such as when interviewees began to cry [31]. Identifiable information was removed and pseudonyms were used. The standards set by the Declaration of Helsinki [32] were consistently adhered to.

### Data analysis

The interviews lasted from 1–2 h (mean 90 min). All the verbatim transcribed interviews were cross-checked against the recordings to verify accuracy. The texts were read repeatedly to gain an overall idea of the fatigue experience [33]. NVivo computer software 10 [34] was used to help organise the dataset and make memos and annotations. In this content analysis [35], the units of analysis were words, sentences, and passages [36] (Table 3).

During the first cycle of the inductive coding process, analytic meaning units were coded and organised into descriptive categories [33]. In the second analytic cycle, the descriptive categories were used to identify patterns. The organisation and reorganisation of codes and subcategorisation was conducted multiple times to identify dimensions, characteristics, and nuances of the phenomenon. The meaning units pertaining to the degree of illness severity were categorised as very severe, severe, moderate and mild [37]. The first and second author repeatedly read through isolated sentences and passages to ascertain coding consistency.

### Rigour

Each person’s illness experience is unique [38], and with very small samples, heterogeneity may be a problem. Thus, the sample included more than 20 participants to obtain sufficient data to capture the range and variations of the fatigue experience. To include participants with different background variables such as work/study status, age, gender, occupation, education level, socioeconomic status, functional level, and symptom burden, a purposive maximum variation sample was selected [25]. Maximum variation aims at “*capturing and describing the central themes that cut across a great deal of variation*” [28]. Different background variables may facilitate rich and broad descriptions and capture core experiences [25]. To avoid predetermined response categories, the interviews were performed with an open approach [28]. Reflective notes were taken immediately after the interviews [25], and analytic memos were recorded in a decision trail [33]. The transcribed interviews and field notes comprised more than 500 pages and provided a rich source of empirical data.

Interviews, verbatim transcriptions, and first cycle coding were performed by the first author, an RN who had previously lectured for patients suffering from ME/CFS at two hospital-based Learning and Mastery Services. Her pre-understanding and views on the topic were explored and made explicit in writing before the study commenced [39]. The second author has published numerous research papers on CFS/ME, whilst the third author’s pre-understanding was influenced by previous research on fatigue in different populations, but she had no experience with PIFS-related fatigue prior to this study.

In both data collection and data analysis, pre-understanding was acknowledged and continuously reflected on and recorded. During the interviews, the researcher adopted an empathic manner to convey an open mind, making the situation comfortable, like a ‘coffee table situation’. To elicit information-rich experiences, the participants were allowed to speak freely in their own words with little interference. To validate the findings, the authors read the texts separately and discussed their understanding throughout the analytical process.

## Results

The qualitative content analysis revealed different themes and subthemes (Table 4) that are presented as follows: 1) the pervasive experience of fatigue; 2) fatigue dimensions; 3) other characteristics of fatigue; and 4) trigger mechanisms. An overview of themes and subthemes is provided in Table 4.

**Table 4:** Themes and subthemes of the fatigue experience in PIFS

Themes	Subthemes
PIFS fatigue as an all-pervasive state	Loss of vitality
Dimensions of fatigue and concomitant symptoms	Awakening fatigue
	Physical fatigue
	Emotional fatigue
	Wired but tired
	Brain fog
	Weird body
Other characteristics of fatigue	Post-exertional malaise and symptom flare-up
	Fluctuations and relapses
	Crashes
	Lack of stamina
	Prolonged restitution time
	Beyond time
	Altered self and body relationship
	Interactive relationship between fatigue dimensions
Mechanisms triggering fatigue and symptom flare-ups	Physical triggers
	Cognitive triggers
	Emotional triggers
	Neurological triggers
	Social triggers
	Financial triggers
	Pressure on oneself and from others

Pseudonyms are used, followed by the participants’ age group. Each category is discussed in further detail, and includes participants’ comments, which have been translated from Norwegian to English and paraphrased.

### Post-infectious fatigue syndrome as an all-pervasive state

While participants had some good days without fatigue, they still did not perceive themselves as healthy. A few had been bed- or housebound for shorter or longer periods and in need of assistance in daily living, whereas others were able to take care of themselves. A minority had been able to work or study part-time. Fatigue was not always present but was provoked or worsened by almost any trigger mechanism, exertions, or energy demanding processes.

#### Loss of vitality

Fatigue was perceived to be a whole-body experience and described as “*all-pervasive*”, “*feeling old*” and being “*out of control*”:

“[W]hen the body stops… the head stops, everything is lost” (Paul, 20s); “[Like] being 95 years old” (Mandy, 20s); “I’m less vital than I was, less alive” (Katherine, 40s).

### Dimensions of fatigue and concomitant symptoms

Fatigue and other symptoms were clustered, and usually fluctuated simultaneously in severity. The symptom intensity of fatigue varied over time and across participants. The participants considered cognitive difficulties more debilitating than physical incapacities; likewise, physical symptoms were perceived easier to tolerate than cognitive ones. The dimensions emerging from the data analysis are described below.

#### Awakening fatigue

Some participants experienced difficulties waking up, but this could vary from day to day. Awakening fatigue was characterised by poor sleep, prolonged awakening process, and feeling drowsy and unrefreshed. Waking up completely could take several hours. Completing routine morning activities was energy demanding and sometimes required rest. The participants described awakening fatigue as “*hibernation”* and “*fatigue worst in the morning*”:

“[I’ve] just been lying in hibernation… [my] body needs time to wake up while I’m pottering about, getting the circulation going” (Fiona, 50s).

#### Physical fatigue

Physical fatigue manifested itself as an “*empty body*” and “*out of order*” because of muscle weakness, lack of energy and difficulties raising energy levels. This was described as “*energy failure”* and “*being very old*”:

“[I feel] like an old battery with sludge at the bottom… it won’t charge… [I am] unable to raise the energy levels” (David, 50s).

#### Emotional fatigue

Many participants felt that they had changed because events handled with patience pre-illness could now evoke emotional reactions. Emotional fatigue comprised irritability, difficulty doing something spontaneously, impatience, frustration, fluctuating temper, lowered stress intolerance, and impaired control. Participants described emotional fatigue as “*having a short fuse”* and *“being in a dark cloud”*:

“When I’m very tired all the emotions follow” (Sue, 20s); “In the evening I become short-tempered… [I am] easily annoyed because my mind doesn’t perform” (Katherine, 40s).

#### Wired but tired

Many patients reported episodes or longer periods of having fatigue and a spinning mind simultaneously. The body and mind appeared to be in an excitable state, difficult to control or stop. This state could follow stressful situations and result in even more fatigue and symptom flare-up. The participants described feeling wired but tired as “*the body was working overtime”* and “*the machinery can’t relax”*:

*“*[A] constant feeling of being wired, but tired for several years” (Brenda, 40s); “My brain continued to tick… like a time bomb” (Lena, 50s).

### Brain fog

All participants felt that their brain function was altered compared with pre-illness. They struggled more or less with cognitive fatigue characterised by word finding difficulties, a reduced ability to concentrate, slow information processing, reduced ability to grasp the meaning of words, forgetfulness, losing trains of thought or story lines in movies and/or books, learning difficulties, reduced intellectual capacity, and confusion. Some had difficulty remembering names or faces, but all experienced memory difficulties. Some could not remember anything from their most severe period. Cognitive fatigue was described as “*brain fog”, “brain not in function”,* and “*Alzheimer’s”*:

“[I] got very confused” (Paul, 20s); “I struggled with people because I had problems expressing myself” (Yvonne, 30s).

The brain’s ability to think coherently, structurally, and perform automatic cognitive processes appeared altered, forcing the participants to think through every step while performing ordinary tasks that required no reflection pre-illness:

“It seemed that thoughts that would previously have been automatic had stopped functioning. The ability to do [things] without having to think was gone. I had to drag them across a relay manually, they didn’t function automatically” (Katherine, 40s).

### Weird body

The description ‘weird body’ includes neurological symptoms such as (a) sensory dysfunction/hypersensitivity, (b) vision and hearing difficulties, and (c) dysregulation of body temperature, paraesthesia, and movement abnormalities.

Sensory fatigue was experienced by all in varying degrees and manifested itself as hypersensitivity or intolerances to noise, light, food, medication, or alcohol. The participants described sensory fatigue as *“too much light*” and “*too much noise*”:

“I couldn’t tolerate… sensations at all” (Claire, 30s), “[I had to take] antidepressants… [it was a] very awful experience… [I] got extremely ill (Yvonne, 30s).

Vision dysfunction was associated with poor eyesight, visual accommodation problems, and eye pain. Hearing difficulties included problems with the discrimination of the major music tones, tone structures falling apart, and problems with tuning out noisy surroundings when engaged in conversation. The ears received auditory stimuli like a satellite dish, ‘a satellite ear’, with a decreased ability to filter out noise and direct hearing focus:

“[V]isual disturbances [things] floated” (Tracy, 30s); “I receive everything as if through a [satellite ear dish]” (Gareth, 50s); “[Music] tones are perceived as noise… with no filter” (Katherine, 40s).

Other complaints included altered temperature control, unsteady gait and stumbling, staggering, difficulties walking in a straight line, twitches, paraesthesia, fainting, light-headedness, poor balance, numbness, and clumsiness. More than half of the participants experienced altered body temperature control, such as hot flushes, cold extremities, chills, and heat/cold intolerance. The participants described neurological fatigue as *“sweats”, “coldness”,* and *“numbness*”:

“[Y]ou start to pull off to one side” (Gary, 30s); “The body becomes weird inside… shivering too… twitches… paraesthesia” (David, 50s); “I’ve become a ‘chilled to the bone’ kind of person” (Gareth, 50s).

Some suffered from symptoms commonly observed in influenza:

“[I had] fever feelings” (Paul, 20s).

### Other characteristics of fatigue and concomitant symptoms

#### Post-exertional malaise and symptom flare-up

PEM was the most prominent feature and was experienced on a regular basis. PEM is characterised by an excessive increase in fatigue and symptom exacerbation following various kinds of exertion, occurring during or immediately after exertion, the next day or several days later. It is a relapse as a result of overexertion and can last for hours, days, and in the most serious cases, for months. Increased fatigue was associated with a lack of energy that resulted in a more severe illness and reduced function or inability to function. The participants perceived PEM as a whole-body experience and described it as “*setbacks”,* “*feeling malaise”* and *“very ill”*:

“[T]he reaction to [exertions] is highly excessive” (Yvonne, 30s); “I don’t struggle right there and then… but the following hours and the next day” (Tom, 50s).

#### Fluctuations and relapses

All participants experienced fluctuations. Variations in fatigue and illness severity occurred as bouts, in a course of day or night, from day to day, during a week, and over months or even years. The participants described unpredictable variations as “*just seem to be cyclic, fluctuating”* and *“days of feeling bad and days of feeling OK, but never 100 %”.* On good days they were “*on the plus side”,* meaning they had extra energy to spend. This resulted in over-expending of energy doing things they had been unable to do on other days, resulting in a bad day the next day. The participants referred to this as “*a price to pay”.* On bad days, “*nothing worked”,* or they were “*exhausted”,* “*out of energy”* and *“feeling bad”:*

“[Things took a] fluctuating course… I have done things and relapsed*”* (Claire, 30s); “I have a lot of pain” (Brenda, 40s); “[Bad] days come without warning” (Grace, 50s).

#### Crashes

A crash, an acute form of PEM, may occur when the body collapses because of an overload of triggers. All participants had experienced acute symptom storms, comprising profound fatigue, severe symptom flare-up, and lack of energy. The symptom storm triggered an acute compulsion to retreat to silent surroundings. Crashes were described as “*hit the wall*”, “*paralysed”* and “*collapsed*”:

“I go into crashes… get paralysed… [need to get] out of the situation” (Katherine, 40s); “[It’s] a sudden need to lie down… close your eyes and just rest!” (Zelda, 40s).

#### Lack of stamina

A lack of stamina comprises a shortage of endurance and fatigability, a lack of simultaneous capacity and warning signs when crossing the capacity limit.

#### Endurance and fatigability

The participants lacked stamina and were unable to sustain activities as they did pre-illness. PIFS appeared to affect the body’s energy supply because the participants suffered from rapid fatigability following different kinds of efforts or strains. Lack of stamina manifested itself as a more or less decreased functional capacity and was described as “*very poor capacity”,* “*no [energy] reserves to call up”,* and *“being completely empty and tired faster”:*

“[I]f there is hardship, I usually try to solve this by adding more coal… but it was empty of coal. No [reserve capacity]” (Katherine, 40s).

#### Reduced simultaneous capacity

Many expressed problems with multitasking. Activities that involved both physical and cognitive efforts simultaneously were handled without problems pre-illness but had now become too demanding. The participants also experienced difficulties if they had to take part in more than one event on the same day:

“I can’t do several things at the same time anymore… If I were to go to the cinema tonight I can’t go to work first; I can only do one of those” (Brenda, 40s).

#### Capacity limit and warning signs

When they were approaching, or exceeding, their body’s functional capacity, various warning signs occurred. Physical, cognitive, psychological, and neurological symptoms indicated over-exertion and crossing a threshold that triggered a total body reaction. The participants typically overestimated their functional capacity and overlooked warning signs, particularly in the first years before they had learned to listen to their body. Warning signs comprised increased fatigue, heartbeat, sweat attacks, feeling faint, muscle pain and weakness, headache, feeling cold, numbness, and a loss of concentration:

“If you cross the threshold, you’ll get worse” (Paul, 20s); “You simply have to listen to the body” (Tom, 20s).

#### Prolonged restitution time

The participants experienced a prolonged restitution period after exertions beyond the capacity limit. Even minimal exertion could require an abnormally long time to recover to pre-exertion functional levels. The time required to regain a higher functional level and less fatigue could vary from hours to days, weeks, months, or years:

“[I] just got worse until I collapsed. [I went from] being completely healthy to staying like this for 3 years, [I was] bedbound for a while… for 3 years I have been in survival mode” (Paul, 20s).

#### Beyond time

Beyond time describes a state of mind when time passes without full consciousness and no awareness of what is going on. Some of the participants experienced this phenomenon at the most serious time of their illness. This appeared to be a state of total resting mode characterised by being in a lethargic, almost coma-like condition in which no cognitive activity occurred. It was described as “*lethargy*”, “*being a zombie*” or “*in limbo*”:

“It seemed like the mind was in a shutdown mode… coma… the concept of time disappeared completely… I had no idea of time” (Yvonne, 30s); “[There were] no thoughts at all… a state of awakening-comatose” (Grace, 50s).

#### Altered self and body relationship

Pre-illness, the participants knew their body’s functional capabilities, and their self was able to instruct the body to perform. The relationship between the self and the body changed as the symptoms became dominant, and the self appeared to lose control. The will to perform was no longer enough to make the body function on demand. All participants wished to perform tasks or participate in activities that the body was unable to handle without triggering PEM:

“It’s not my willpower that governs… the symptoms govern daily life” (Mandy, 20s).

#### Interactive relationship between fatigue dimensions

Increased severity in one fatigue dimension could lead to increased severity of another, indicating an interactive relationship. Hence, increased cognitive fatigue could be followed by an increase in physical fatigue or vice versa:

“When you feel the body is better, it’s followed by improvement in other [cognitive symptoms] too… I got physically better before I got cognitively better, so it’s linked together” (Andrew, 40s).

### Mechanisms that trigger fatigue and symptom flare-up

Triggers that emerged from the participants’ accounts encompassed personal, physical, cognitive, emotional, social, financial, and neurological/sensory exertions or energy draining processes. What the participants were able to do or tolerate before their capacity limit was reached depended on the severity of their fatigue at a given time.

#### Physical triggers

The intensity of physical triggers or energy demanding tasks provoking increased fatigue ranged from minor, such as talking or taking care of basic needs, to normal activities in everyday life, exercise or recreational activities. In *very severely affected* bed- or housebound participants, triggers such as speaking, sitting or standing upright, moving around, and taking care of oneself could increase fatigue:

“You even get tired from speaking… from sitting” (Wanda, 50s).

In *severely affected* participants*,* triggers such as telephone calls, shopping, preparing meals, carrying things, or walking short distances could increase fatigue:

“I watched my son make his lunch pack and went back to bed to lie down… I can’t make dinner or shop or carry a bag” (Alice, 30s).

In *moderately affected* participants*,* triggers such as homemaking, taking care of children, part time work/studying, travelling, or vacation could increase fatigue:

“[A]fter trying to work for 14 days I became much worse” (Yvonne, 30s); “I tried to exercise and collapsed like a bunch of broccoli” (Sue, 20s).

In *mildly affected* participants*,* triggers such as too much work or study, strenuous physical exertions, exercise and mountain walks could increase fatigue and provoke PEM:

“[I] walk… on Thursdays. Fridays I’ve been quite ill” (Andrew, 40s); “[T] he holiday travel exertion was too strenuous… [I] collapsed (Wanda, 50s).”

#### Cognitive triggers

All of the participants reported cognitive triggers such as reading, writing, and computer work, watching television, and listening to the radio. TV programs with fast changing pictures and complicated issues such as calculation or learning new things triggered fatigue:

“The brain becomes worse from all the concentration, whether it’s watching TV or reading. Computer work is definitively the worst, as I begin to cold sweat and feel dizzy” (Paul, 20s).

#### Emotional triggers

Many reported emotional triggers such as watching films with emotional content or being in a physically and mentally stressful situation. When the participants had many things waiting to be accomplished, it was perceived as very stressful, and the fatigue increased:

“If I watched films, emotional things, like something that touched me I just had to turn such things off” (Amanda, 40s).

#### Neurological triggers

Sensory trigger mechanisms such as sounds, lights, fast moving surroundings, commotion, and intolerances could make the participants more fatigued or increase symptom intensity. Being at shopping malls or airports, where multiple sensory triggers occurred simultaneously, could suddenly provoke exacerbation:

“[I’ve] been shopping… suddenly it is enough” (Zelda, 40s).

#### Social triggers

Social triggers such as being with other people, participation in cultural activities, and taking part in everyday life could provoke increased fatigue and PEM. Too many stimuli from the environment or the situation might provoke symptom exacerbation:

“I become more fatigued by being with other people” (Emma, 40s); “When attending concerts or shows… I became completely destroyed” (Lena, 50s).

#### Financial triggers

Financial strains were common. Illness severity left the participants unable to earn enough money to provide for themselves or their family. Strained household finances and the lack of support from the social security system and/or insurance company increased their fatigue. Pushing oneself to work often resulted in the inability to work at all:

“[Economic problems] worsened my fatigue” (Wanda, 50s); “[T]he welfare system that’s supposed to help you… clearly [inhibits improvement]… the energy you should have used to become well … you spend being worried” (Paul, 20s).

#### Pressure on oneself and from others as triggers

The participants wanted to continue with their pre-illness life despite this being counterproductive. The pressure put on oneself or from others acted as triggers. All of the participants expressed “*I pushed myself too hard*”:

“I went on at the same pace as I did before I got ill” (Grace, 50s); “I really regret that I forced myself to be active… this was wrong but my general practitioner insisted” (Yvonne, 30s).

## Discussion

### Findings

The general nature of post-infectious fatigue was perceived to be a fluctuating, whole body, all-pervasive state that interfered with all aspects of life. All participants described fatigue as a pervasive lack of energy and stamina and experienced unpredictable symptom flare-ups that contributed to functional disability. These findings are consistent with previous reports [11, 17]. As also noted by Cohn [16], there was a striking coherence in the participants’ accounts.

Three dimensions of fatigue were identified: awakening, physical, and emotional. Physical and mental fatigue have been described previously [11]. ‘Emotional fatigue’ in our study has similarities with ‘mood disturbance’ [12] and ‘emotional distress’ [40]. However, this study uncovered a dimension termed ‘awakening fatigue’, which has not been previously reported. This is also the first time that ‘beyond time’ is highlighted as a characteristic of fatigue. It appears to occur during the most severe periods and may be a type of vegetative state.

Fatigue coexisted with symptom dimensions such as feeling “wired but tired” and experiencing “brain fog” and “weird” symptoms. Earlier qualitative and quantitative studies in ME/CFS have identified similar dimensions: ‘wired’ and ‘brain fog’ [13, 41], ‘neurocognitive impairment’ [12] and ‘neurological impairment’ [42]. Furthermore, ‘visual impairment’ [43] and ‘flu-like’ symptoms are found in CFS [13, 42]. In our study, ‘visual impairment,’ ‘neurological dysfunction’ and ‘flu-like’ were central aspects of ‘weird body’.

Comparing dimensions between studies is challenging because of heterogeneity and differences in illness duration, use of labels, and content of dimensions [9, 44], but there are several similarities. Illness duration in other study samples varies considerably. All participants in our sample, however, developed PIFS in the aftermath of a known *G. lamblia* infection and remained ill when the interviews were conducted 4 years later. Because they were exposed to the same precipitating cause and had the same illness duration, this study provides new insights into the nature and course of PIFS-related fatigue and its impact on the lives of the patients.

An important finding was the interactive nature of the dimensions, the co-variation of the intensity of fatigue and the concomitant symptoms. This is in line with the findings of Arroll and Senior [15] who studied ME/CFS patients. Several of the concomitant symptom dimensions uncovered in our study have also been identified in CFS patients [45].

PIFS was triggered by several mechanisms followed by an abnormal time to recover to pre-exertion levels. Minor triggers could still provoke PEM, symptom flare-ups, and relapses. The intensity of the fatigue and the concomitant symptoms varied considerably, were difficult to control, and depended on several factors.

Characteristics such as PEM, crashes, and prolonged restitution time have been described elsewhere [4, 15], as have fluctuations [11, 15], unpredictability [15, 18], altered relationship between body and self [11, 18], interaction between dimensions [11], and excessive increase in fatigue in response to minor activities [12]. Energy was a resource that could be depleted, but the energy building process was slow, and energy levels did not return to pre-illness levels.

Physical and mental exertions have been previously identified as trigger mechanisms [15]. This study uncovered several additional mechanisms such as emotional, neurological, social, financial, and pressure put on oneself or from others. Continuing pre-illness lifestyles resulted in crossing the capacity limit, thus provoking increased fatigue and PEM. This counterproductive method of handling the illness has been noted earlier [11] and may result from lack of knowledge or awareness of the underlying mechanisms that trigger or increase fatigue [15].

The condition evolved over the 4 years following the *Giardia*-induced infection that triggered PIFS, but the evolution did not occur in the same way for all participants. Although each participant was healthy pre-illness and the trigger mechanism was the same, the range of improvement varied. None had resumed pre-illness personal or professional functional levels.

The treatment of fatigue must be individually tailored [46]. RNs and other clinicians should recognise fatigue as something distinct from depression and lack of motivation [47]. This study provides a broad in-depth understanding of the debilitating, complex nature of fatigue. Knowledge of its different dimensions, characteristics, and trigger mechanisms makes it possible to grasp the complexity and understand the consequences for the sufferer.

### Strengths and limitations

The strengths of this study are the relatively large and strategic maximum variation sample, a well-defined population with a known precipitating cause and the open-ended interview approach. There are also several limitations. The sample included only persons well enough to participate, ruling out the most severely affected. Furthermore, this cohort fell ill following a confirmed gastrointestinal infection and might therefore differ from samples with insidious onset and/or other infectious agents. People affected with PIFS/ME/CFS around the world constitute a heterogeneous group. As most cases are triggered by infectious agents, the findings may often be transferable, but may not be fully applicable to cases triggered by various non-infectious precipitating factors. Finally, several participants reported substantial gaps in their memory of the preceding 4 years. Experiences are personal and subject to recall bias, so some aspects of fatigue may have been missed.

### Implications for nursing practice, education and research

Patients with PIFS and the general population have different perceptions of fatigue [13]. Thus, RNs and other health practitioners may have difficulties in recognising and understanding the patients’ fatigue experience. Patients’ perceptions are multidimensional, multifaceted, and heterogeneous in nature, and fatigue manifests itself in different ways. Because these patients are often misunderstood, listening to their language and illness experience serves as a diagnostic component. The findings of this study provide RNs with vocabulary and knowledge, making it easier for them to develop awareness and symptom recognition. A common vocabulary may facilitate communication and mutual understanding. Nurses need to educate themselves to be able to recognise PIFS. Greater knowledge may contribute to more accurate observations and thus more appropriate nursing care. This in turn may help alleviate suffering and assist sufferers in self-management, promoting a path to improvement. Further exploration of fatigue in PIFS is required because the condition is heterogeneous. Future research should explore gender differences and fatigue experiences in severely affected house- or bedbound individuals. Longitudinal studies examining whether or how fatigue and concomitant symptoms occur at the onset or in the early or chronic phases are also needed.

## Conclusions

Fatigue is not fully understood [13] and obtaining in-depth knowledge of the complex characteristics of PIFS fatigue appears fruitful. Dimensions, characteristics, and trigger mechanisms may provide insight and a language to convey experiences to families, friends, and health personnel [15]. Because early diagnosis may lead to a better prognosis [4], it is important for RNs to increase their understanding and knowledge of fatigue, thereby increasing their likelihood of recognising its symptoms and offering appropriate nursing care.
